# HOXA1 participates in VSMC-to-macrophage-like cell transformation via regulation of NF-κB p65 and KLF4: a potential mechanism of atherosclerosis pathogenesis

**DOI:** 10.1186/s10020-023-00685-8

**Published:** 2023-08-01

**Authors:** Zhiyang Han, Haidi Hu, MingZhu Yin, Yu Lin, Yan Yan, Peng Han, Bing Liu, Bao Jing

**Affiliations:** 1 Department of Vascular Surgery, The First Affiliated Hospital of Harbin Medical University, No. 23, Youzheng Street, Harbin, 150001 Heilongjiang China; 2 Department of General and Vascular Surgery, Shengjing Hospital of China Medical University, Shenyang, 110001 Liaoning China; 3 Department of Dermatology, Xiangya Hospital Central South University, Changsha, 410008 Hunan China; 4 Human Engineering Research Center of Skin Health and Disease, Changsha, 410008 Hunan China

**Keywords:** HOXA1, Atherosclerosis, VSMC, RelA, KLF4

## Abstract

**Background:**

Macrophage-like transformation of vascular smooth muscle cells (VSMCs) is a risk factor of atherosclerosis (AS) progression. Transcription factor homeobox A1 (HOXA1) plays functional roles in differentiation and development. This study aims to explore the role of HOXA1 in VSMC transformation, thereby providing evidence for the potential mechanism of AS pathogenesis.

**Methods:**

High fat diet (HFD)-fed apolipoprotein E knockout (ApoE^−/−^) mice were applied as an in vivo model to imitate AS, while 1-palmitoyl-2-(5-oxovaleroyl)-sn-glycero-3-phosphocholine (POV-PC)-treated VSMCs were applied as an in vitro model. Recombinant adeno-associated-virus-1 (AAV-1) vectors that express short-hairpin RNAs targeting HOXA1, herein referred as AAV1-shHOXA1, were generated for the loss-of-function experiments throughout the study.

**Results:**

In the aortic root of AS mice, lipid deposition was severer and HOXA1 expression was higher than the wide-type mice fed with normal diet or HFD. Silencing of HOXA1 inhibited the AS-induced weight gain, inflammatory response, serum and liver lipid metabolism disorder and atherosclerotic plaque formation. Besides, lesions from AS mice with HOXA1 knockdown showed less trans-differentiation of VSMCs to macrophage-like cells, along with a suppression of krüppel-like factor 4 (KLF4) and nuclear factor (NF)-κB RelA (p65) expression. In vitro experiments consistently confirmed that HOXA1 knockdown suppressed lipid accumulation, VSMC-to-macrophage phenotypic switch and inflammation in POV-PC-treated VSMCs. Mechanism investigations further illustrated that HOXA1 transcriptionally activated *RelA* and *KLF4* to participate in the pathological manifestations of VSMCs.

**Conclusions:**

HOXA1 participates in AS progression by regulating VSMCs plasticity via regulation of NF-κB p65 and KLF4. HOXA1 has the potential to be a biomarker or therapeutic target for AS.

**Supplementary Information:**

The online version contains supplementary material available at 10.1186/s10020-023-00685-8.

## Introduction

Atherosclerosis (AS) is the fundamental pathological state of many vascular disorders, and a leading cause of fatal heart attacks and strokes worldwide (Falk 2006; Frostegård 2013). AS begins with the infiltration and retention of apolipoprotein B that contains lipoproteins in the subendothelial region of the artery, and processes with the recruitment and accumulation of inflammatory cells, extracellular matrix and lipids (Skålén et al. 2002). Macrophages are the major leukocyte subset in the subendothelial area of blood vessels. During AS development, macrophages could become foam cells by ingesting residual lipoproteins, and trigger pro-inflammatory responses by oxidation modification or other stimulus (Chistiakov et al. 2017). Then, the modified endothelial smooth muscle cells or other sources of cells migrate and enter into the intima, leading to the formation of collagenous fibrous cap that coats lipid-rich plaque core (Harman and Jørgensen 2019). The plaques develop in the context of oxidative lipids, foam cells, persistent inflammatory stimulation and defective clearance of dead cells (Castaño et al. 2020). Over time, the integrity of the collagenous fibrous cap may be compromised, resulting in plaque rupture and acute occlusive thrombosis.

One of the fundamental bioprocesses of AS progression is the functional variation of vascular smooth muscle cells (VSMCs) (Chistiakov et al. 2015). VSMCs are one of the major cell types existing at all stages of an atherosclerotic plaque (Basatemur et al. 2019). The historical view of VSMCs in AS pathogenesis is that the contractile VSMCs undergo phenotypic conversion to proliferative synthetic cells, generating extracellular matrix to form the fibrous cap, to cover the necrotic core and to stabilize the plaques (Bennett et al. 2016; Mitchell and Sidawy 1998). However, recent lineage tracing experiments in apolipoprotein E knockout (ApoE^−/−^) mice reveal that VSMCs are more plastic than previously recognized and are able to adopt alternative phenotypes, including phenotypes resembling macrophage-like, foam cell-like, osteochondrogenic-like, and mesenchymal stem cell-like cells, which makes positive or negative contribution to disease progression (Chappell et al. 2016; Gomez et al. 2018). Some in vitro studies also documented that under the action of cholesterol, VSMC could transdifferentiate into macrophage-like cells, with ensuing formation of lipid-filled foam cells (Rong et al. 2003; Vengrenyuk et al. 2015). Unlike monocyte-derived macrophages with a powerful phagocytic ability, these VSMC-derived macrophage-like cells have a much weaker phagocytic ability and tend to accumulate in plaques, leading to high absorption of lipids and deficient efferocytotic removal of apoptotic cells and debris, which further accelerates the formation of plaques (Harman and Jørgensen 2019). However, current therapeutic approaches to vulnerable plaques are very limited and the mechanisms involved in VSMC phenotypic transition are not well understood. Therefore, deciphering the underlying mechanisms in plaque development has important implications for understanding AS pathogenesis and developing therapeutic strategies.

The homeobox (HOX) gene family encode a series of homeodomain-containing transcription factors that participate in mammalian tissue growth and differentiation (Mohankumar et al. 2007; Primon et al. 2019). Homeobox A1 (HOXA1), the first HOX gene found to be associated with gastrulation, is critical for the developmental program that coordinates the behaviour of cells during embryogenesis (Gouti et al. 2011; Makki and Capecchi 2010; McNulty et al. 2005). However, the misexpression of HOXA1 in differentiated cells could make it become an oncogene to participate in cancer development (Bitu et al. 2012; Xiao et al. 2014). In particular, our previous work showed that overexpression of miR-99a-5p could ameliorate atherosclerotic lesions in high-fat diet (HFD)-fed ApoE^−/−^ mice, accompanying with a decrease in HOXA1 expression (Han et al. 2019). Another work by Yu et al. also documented that the mechanism by which lncRNA ROR influences AS involves let-7b-5p/HOXA1 axis (Yu et al. 2021). Notably, an earlier study reported that HOXA1 is a target of miR-10a, and HOXA1 is up-regulated in the athero-susceptible regions (inner aortic arch and aorto-renal branches) than elsewhere (Fang et al. 2010). Nevertheless, the explicit role of HOXA1 in AS progression remains undefined.

As a transcriptional factor, HOXA1 could bind with a gene’s promoter region to regulate its expression, thereby participating in the regulation of disease progression (Chen et al. 2022). Nuclear factor (NF)-κB was discovered long time ago and has become as a master regulator of inflammation and immune homeostasis, playing a central role in diseases comprising a significant inflammatory component including AS (Mitchell and Carmody 2018). Activation of NF-κB is a critical event in triggering VSMC functional alterations (Farina et al. 2022; Yeh et al. 2019). In addition, krüppel-like factor 4 (KLF4) is also an essential regulator of AS development. The KLF4-dependent phenotypic modulation of VSMCs is vital for the atherosclerotic plaque pathogenesis (Shankman et al. 2015). Notably, with JASPAR’s prediction, we noticed there are binding regions of HOXA1 in both NF-κB RelA’s promoter and KLF4’s promoter. Therefore, HOXA1 is likely to be involved in the regulation of VSMC alterations in AS progression by regulating NF-κB and KLF4. In the current study, we experimentally verified the functional role of HOXA1 in AS progression in both in vivo and in vitro models*,* and investigated the involved molecular mechanisms.

## Materials and methods

### Construction of recombinant adeno-associated virus-1 (AAV1) vectors

Short-hairpin RNAs (shRNAs) targeted to mouse or human HOXA1 and its scramble control (shNC) were synthesized. The sequences of shRNAs were: mus shHOXA1-1: GAATCATCTGAGAAATCTAGCTTCAAGAGAGCTAGATTTCTCAGATGATTCTTTTT; mus shHOXA1-2: AGTACATTCACCACTCATATGTTCAAGAGACATATGAGTGGTGAATGTACTTTTTT; homo shHOXA1-1: CTCGGACCATAGGATTACATTCAAGAGATGTAATCCTATGGTCCGAGTTTTT; homo shHOXA1-2: GCTGTTTACTCTGGAAATCTTCAAGAGAGATTTCCAGAGTAAACAGCTTTTT; shNC: TTCTCCGAACGTGTCACGTTTCAAGAGAACGTGACACGTTCGGAGAATTTTTT; AAV1 vectors were recombined with restriction enzyme digestion (HindIII and XhoI) to carry shRNA coding sequences. Virus particles were obtained from HEK293 cells.

### Establishment of a mouse model of AS

To investigate the expression of HOXA1 in atherosclerotic mice, 8-week-old wild-type (WT) male mice and ApoE^−/−^ male mice on the C57BL/6 background were used in this study. ApoE^−/−^ mice were fed with high-fat diet (HFD) to induce AS. WT mice with normal diet (ND) feeding or HFD were allocated as controls. Twelve weeks after HFD feeding, mice were euthanized by CO_2_ inhalation. Aortic root and liver tissues were collected for examination.

To investigate the role of HOXA1 in AS progression, 8-week-old ApoE^−/−^ male mice were injected with 5 × 10^10^ vector genome of AAV1-shNC, AAV1-shHOXA1-1 or AAV1-shHOXA1-2 via tail vein and then received HFD feeding for 12 weeks. Blood samples, aortic and liver tissues were collected for examination.

### Western blot

Total proteins were extracted using RIPA lysis buffer (Beyotime Institute of Biotechnology, Shanghai, China). Nuclear and cytoplasmic proteins were extracted using nuclear and cytoplasmic protein extraction kit (Beyotime). Protein concentrations were determined by BCA assay (Beyotime). Twenty micrograms of proteins were separated by 8%, 10%, or 12% SDS-PAGE (Beyotime), and then transferred to polyvinylidene difluoride (Thermo Fisher Scientific, Pittsburgh, PA, USA) membranes. The membranes were blocked with 5% bovine serum albumin solution (Biosharp life sciences, Hefei, China) at room temperature for 1 h and then incubated with primary antibodies including anti‐HOXA1 (1:500 dilution; Cat. No. DF3187, Affinity Biosciences, Cincinnati, OH, USA), anti‐VCAM1 (1:2000 dilution; Cat. No. A0279, ABclonal Biotechnology, Wuhan, China), anti‐MMP2 (1:1000 dilution; Cat. No. 10373-2-AP, Proteintech Group, Rosemont, IL, USA), anti‐p65 (1: 1000 dilution; Cat. No. A19653, ABclonal), anti‐phospho-p65 (1:1000 dilution; Cat. No. AP0475, ABclonal), anti‐KLF4 (1: 1000 dilution; Cat. No. A13673, ABclonal), anti-Histone H3 (1:500 dilution; Cat. No. 17168-1-AP, Proteintech), and anti-β-actin (1:2000 dilution; Cat. No. 60008-1-Ig, Proteintech) at 4 °C overnight. After washed with TBST, the membranes were incubated with secondary antibodies including HRP-labelled Goat anti-Mouse IgG (1:10,000 dilution; Cat. No. SA00001-1, Proteintech) or HRP-labelled Goat anti-Rabbit IgG (1: 10,000 dilution; Cat. No. SA00001-2, Proteintech). The membranes were washed again and then visualized with an ECL kit (Seven-sea Pharmtech, Shanghai, China). β‐actin was used as the control for total proteins and Histone H3 was used as the control for nuclear fractions.

### Immunofluorescence

The frozen sections of thoracic aortas and aortic roots were subjected to antigen retrieval and blocked with goat serum. Afterwards, the sections were incubated with anti-α-SMA antibody (1:200 dilution; Cat. No. sc-53142, Santa Cruz, Santa Cruz Biotechnology, Dallas, TX, USA), anti-HOXA1 antibody (1:100 dilution; Cat. No. DF3187, Affinity), anti-KLF4 antibody (1:50 dilution; Cat. No. 11880-1-AP, Proteintech), or anti-Mac3 antibody (1:200 dilution; Cat. No. 66301-1-Ig, Proteintech) overnight at 4 °C, followed by incubation with Cy3-labeled goat anti-mouse IgG (1:200 dilution; Cat. No. A0516, Beyotime) or FITC-labelled goat anti-mouse (1:200 dilution; Cat. No. A0562, Beyotime) at room temperature for 1 h in the dark. The sections were then counter-stained with DAPI (Aladdin Reagents, Shanghai, China) to identify cell nuclei and imaged with an Olympus BX53 microscope.

### Real-time quantitative polymerase chain reaction (real-time qPCR)

Total RNAs in thoracic aortas or cultured VSMCs were extracted using an RNApure high-purity total RNA rapid extraction kit (BioTeke Corporation, Beijing, China) according to the manufacturer’s instructions. The first-strand complementary DNAs were synthesized with oligo(dT)_15_ as primer using M-MLV reverse transcriptase (Takara Biomedical Technology, Beijing, China). Real-time quantitative polymerase chain reaction (qPCR) was performed using SYBR Green I (BioTeke) in Bioneer Exicycler 96 instrument. The sequences for primers were: Homo *HOXA1*, 5’-CGCTCCCGCTGTTTACTC-3’ and 5’-AGGCTCTGGTGCTCCTGTCC-3’; Homo NF-κB *RelA* (p65), 5’-GGGGACTACGACCTGAATG-3’ and 5’-GGGCACGATTGTCAAAGAT-3’; Homo *KLF4*, 5’-CGAACCCACACAGGTGAGAA-3’ and 5’-TACGGTAGTGCCTGGTCAGTTC-3’; Homo *MAC2*, 5’-ATGATGCGTTATCTGGGTCT-3’ and 5’-GGTGGCACTTGGCTGTC-3’; Homo *MAC3*, 5’-CCAGAAGCTGGAACCTA-3’ and 5’-CTGCCTGTGGAGTGAGT-3’; Homo *ABCA1*, 5’-TCACCACTTCGGTCTCC-3’ and 5’-CCACCTTCATCCCATCT-3’; Homo *ACTA2* (α-SMA), 5’-GGGGTGATGGTGGGAATG-3’ and 5’-GCAGGGTGGGATGCTCTT-3’; Homo *MYH11*, 5’-CAGGATAGGGCAGAGCAA-3’ and 5’-GCCAAGTAGCCACGACAC-3’; Homo *CNN1*, 5’-CCACCCTCCTGGCTTTG-3’ and 5’-ATGATGTTCCGCCCTTCT-3’; Mus *HOXA1*, 5’-AAGCAGAAGAAGCGTGAGA-3’ and 5’-GTGGGAGGTAGTCAGAGTGTC-3’.

### Lipids measurement

Total cholesterol (TC), triglyceride (TG), low-density lipoprotein cholesterol (LDL-C), and high-density lipoprotein cholesterol (HDL-C) levels in the serum and liver were examined using commercially available kits (Nanjing Jian Cheng Bioengineering Institute, Nanjing, China) according to the manufacturer’s instructions.

### Histopathological examination

The thoracic aortas, aortic roots, or the livers were carefully isolated and fixed in 4% paraformaldehyde. The fixed thoracic aortas, aortic roots, or the livers sections were stained with oil red-O (Sigma-Aldrich, St. Louis, MO, USA) for examination of lesion lipid content. The aortic root sections were subjected to hematoxylin (Solarbio Science & Technology, Beijing, China) and eosin (Sangon Biotechnology, Shanghai, China) staining for examination of atherosclerotic lesions, and picrosirius red staining (Solarbio) for examination of collagen deposition. Staining images were captured using an Olympus BX53 microscope.

### Apoptosis assessment in plaques

Apoptotic cells in plaques were assessed using an In Situ Cell Death Detection Kit (Roche, Nutley, NJ, USA) following the manufacturer's instructions. The staining results were imaged with an Olympus BX53 microscope.

### Inflammatory cytokines measurement

Tumour necrosis factor-α (TNF-α), interleukin (IL)-1β and IL-6 levels in thoracic aorta, and TNF-α and matrix metalloproteinase 2 (MMP2) levels in VSMC culture, were measured using corresponding enzyme-linked immunosorbent assay (ELISA) kits (USCN Life Science, Wuhan, China) according to the manufacturer’s instructions.

### Electrophoretic mobility shift assay (EMSA)

The 5′ biotin-labelled mouse *RelA* promoter DNA probe and human *RelA* promoter DNA probe were synthesized by Sangon. Nuclear proteins in thoracic aorta plaques or in cultured VSMCs were extracted using nuclear protein extraction kit. Protein concentrations were determined by BCA assay. Electrophoretic mobility shift assays were performed with a chemiluminescent EMSA Kit (Beyotime). Negative control and competitor control were adopted as references according to the manufacturer’s instructions.

### Human VSMCs culture, treatment, adeno-associated virus infection and plasmid transfection

Human VSMCs were purchased from ScienCell (Beijing, China) and cultured in SMC culture medium (ScienCell). Human 293 T cells were purchased from Zhong Qiao Xin Zhou (Shanghai, China) and cultured in DMEM medium (Hyclone, Logan, UT, USA) supplemented with 10% fetal bovine serum (Bioind, Kibbutz Beit-Haemek, Israel).

VSMCs were infected with AAV1-shNC, AAV1-shHOXA1-1, or AAV1-shHOXA1-2 at a multiplicity of infection of 150 following the group information. Twenty-four hrs after adeno-associated virus infection, the supernatant was changed with fresh complete medium. For detection of infection efficiency, cells were cultured for another 48 h and real-time qPCR and western blot were performed to detect infection efficiency the expression of HOXA1. For biological examinations, cells were cultured for another 24 h and then treated with 30 μg/mL 1-palmitoyl-2-(5-oxovaleroyl)-sn-glycero-3-phosphocholine (POV-PC, Aladdin) for 24 h.

For the rescue experiments, VSMCs were infected with adeno-associated viruses for 24 h and then the supernatant was changed with fresh complete medium. Cells were cultured for another 24 h and then transfected with RelA or KLF4 overexpression plasmid for 24 h. POV-PC was added to the medium together with the plasmids.

### Detection of lipid accumulation in VSMCs

After POV-PC treatment for 24 h, VMSCs were fixed with 4% paraformaldehyde and stained with 0.5% oil red-O staining buffer. The staining results were imaged with an Olympus BX53 microscope.

### Flow cytometry

After POV-PC treatment for 24 h, VMSCs were collected and incubated with anti-human CD68 FITC (0.125 μg/10^6^ cells; eBioscience, San Diego, CA, USA) for 30 min in the dark. A flow cytometry was performed immediately in NovoCyte flow cytometer (ACEA Biosciences, San Diego, CA, USA).

### Luciferase reporter assay

Full length of homo HOXA1 was ligated into the plasmid expression vector pcDNA3·1 with restriction enzyme digestion (HindIII and XhoI). Empty vector was used as a negative control. The *RelA* promoter fragment (− 2000/ + 1) or *KLF4* promoter fragment (− 2000/ + 1) was amplified by PCR and ligated into the pGL3-Basic vector. 293 T Cells grown at a confluence of 70% were transfected with pcDNA3·1 plasmid and pGL3-Basic vector. Forty-eight hours after transfection, the Firefly value and Renilla value in cells were detected and fold change was calculated as Firefly/Renilla.

### Chromatin immunoprecipitation (ChIP)-qPCR

ChIP assay was performed using a ChIP assay kit (Wanleibio, Shenyang, China) according to the manufacturer’s instructions. Briefly, cells were cross-linked with 1% formaldehyde for 10 min and the crosslinking reaction was stopped with 0.125 M glycine. Chromatin was sonicated to generate 500–1000 bp DNA fragments. Immunoprecipitation of HOXA1 from VSMCs was performed using anti-HOXA1 (Cat. No. sc-293257, Santa Cruz) and anti-IgG with Protein G agarose beads. The complexes were de-crosslinked with 5 M NaCl and treated with Proteinase K. The eluted DNA fragments were then subjected to qPCR using primers specific to the *RelA* or *KLF4* promoter or the non-binding regions as negative controls. Positive and negative genomic regions for HOXA1 binding were amplified using the following primers: positive region (− 1840 to − 1542 upstream of TSS site) of *RelA* (F) CCACTTTGGGAGGCTGAG and (R) TGGGACTACGGGAGCACA; negative region (− 794 to − 597 upstream of TSS site) of *RelA* (F) GAGGTCCAGGGCAAACAC and (R) GCGCCAGCTTACGATACA; positive region (− 1383 to − 1178 upstream of TSS site) of *KLF4* (F) CTGAGCCGAAGGAACGAG and (R) TGAGCCACTGCCTGTAAT; negative region (− 1010 to − 828 upstream of TSS site) of *KLF4* (F) CAGGAGAATCGCTTGGAC and (R) GAAGAGGGACTGTGAGGC.

### Oligonucleotide pull-down

The biotinylated double-stranded oligonucleotides specific for the *RelA* or* KLF4* promoter or the non-specific oligonucleotides were incubated with streptavidin-conjugated agarose beads. Then the oligo/streptavidin-conjugated beads were added to each reaction. After incubation, beads were washed and the retrieved protein was collected. The affinity-purified proteins were eluted with protein elution buffer. The elute proteins were then subjected to Western blot analysis.

### Image quantification

All image quantifications were conducted with the Image J software by two independent researchers blinded with the group information. Quantifications of the plaque lipid (oil-red O stained sections), necrotic core (H&E stained sections), and collagen content (PSR stained sections) were defined using the colour thresholding option in Image J. In each case, the positive area was defined as a percentage of total plaque area in each aortic root section. In addition, quantification of the immunofluorescence images was conducted by counting the positive cell number per field.

### Statistical analysis

All experiments were performed at least in triplicate. All data were presented as mean ± standard deviation (SD). GraphPad Prism (version 8; San Diego, CA, USA) was applied to analyse all Data. Gaussian distribution of the residuals within the groups was evaluated using the Shapiro–Wilk test. Homogeneity of variance was assessed using Brown-Forsythe test. Data with residuals that met Gaussian distribution and with equal SD were analysed with ordinary one-way ANOVA followed by Tukey's multiple comparison test. Data with residuals that met Gaussian distribution and with unequal SD were analysed with Brown-Forsythe and Welch ANOVA followed by Games-Howell's multiple comparisons test. Data with residuals that not met the Gaussian distribution were analysed with Kruskal–Wallis test followed by Dunn's multiple comparisons test. ^*^p < 0.05, ^**^p < 0.01, and ^***^p < 0.001 were all considered statistically significant.

## Results

### HOXA1 expression is increased in the aortic root of AS mice

**Fig. 1:**
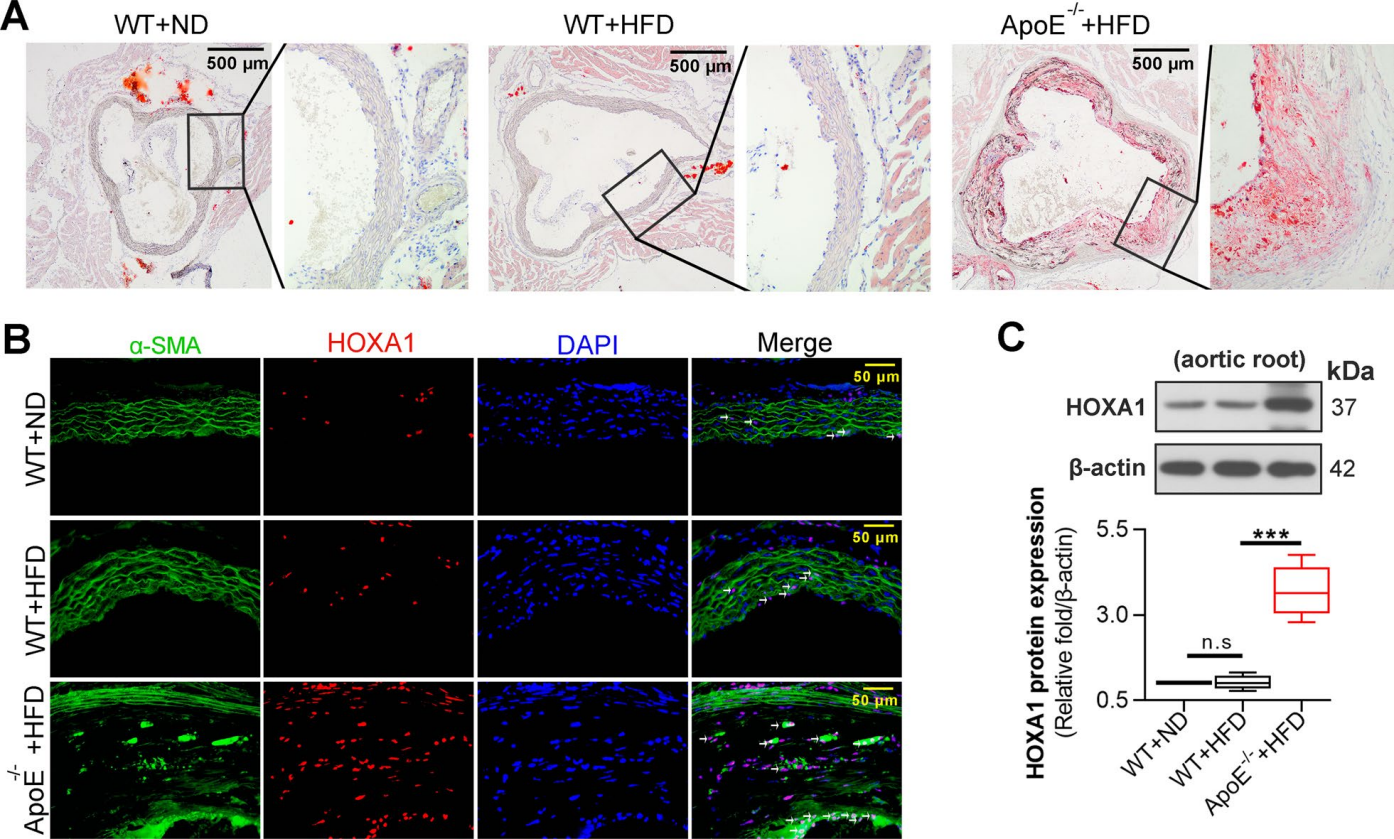
HOXA1 expression is increased in the aortic root of AS mice. **A** Oil red-O staining for the aortic root of wild-type mice with ND, wild-type mice with HFD, or ApoE^−/−^ mice with HFD. Scale bar = 500 μm (40 ×). **B** Immunofluorescence double staining for HOXA1 (red) and α-SMA (green) in each group. DAPI (blue) was used for nucleus staining. Scale bar = 50 μm (400 ×). **C** Western blot for HOXA1 expression in aortic root of wild-type or HFD-fed ApoE^−/−^ mice. Data are expressed as mean ± SD (6 animals/group). Ordinary one-way ANOVA followed by Tukey's multiple comparison test was used to calculate the *P* value in panel **C** ^***^P < 0.001

To ensure the expression pattern of HOXA1 in the context of AS, we established a mice model by feeding ApoE^−/−^ mice with HFD for 12 weeks, whereas wild-type mice fed with normal diet (ND) or HFD were allocated as controls. Oil red O staining indicated that significant plaque formation occurred in the aorta root of ApoE^−/−^ mice that fed with HFD, whereas basically no plaques were observed in the aorta root of WT + ND or WT + HFD mice, implying that only ApoE^−/−^ mice with HFD developed AS (Fig. 1A). Results of immunofluorescence double staining in the aortic root showed the presence of many double-positive HOXA1/α-SMA cells in ApoE^−/−^ + HFD group, while a very small number of double-positive HOXA1/α-SMA cells were seen in WT + ND or WT + HFD group (Fig. 1B). Results of Western blot confirmed the overall enhancement of HOXA1 expression in the aortic root of ApoE^−/−^ + HFD mice. These findings suggested that HOXA1 expression showed an upward trend in AS lesions, and was associated with VSMCs (Fig. 1B, C).

### Silencing of HOXA1 attenuates AS-associated weight gain, aortic lesions and inflammation in mice

To evaluate the functional role of HOXA1 in AS-associated characteristics, we knocked down HOXA1 expression with AAV1-mediated delivery system in ApoE^−/−^ mice to perform loss-of-function experiments. Two AAV1-shHOXA1 vectors, herein referred as AAV1-shHOXA1-1 and AAV1-shHOXA1-2, were generated to reduce the off-target effects. AAV1-shNC vector was applied as a non-specific control. The efficiency of HOXA1 silencing was determined by real-time qPCR and Western blot. The results showed that AAV1-shHOXA1-1/2 intervention successfully knocked down HOXA1 at both mRNA and protein levels in the vessels of mice (Additional file 1: Fig. S1A, B).

**Fig. 2:**
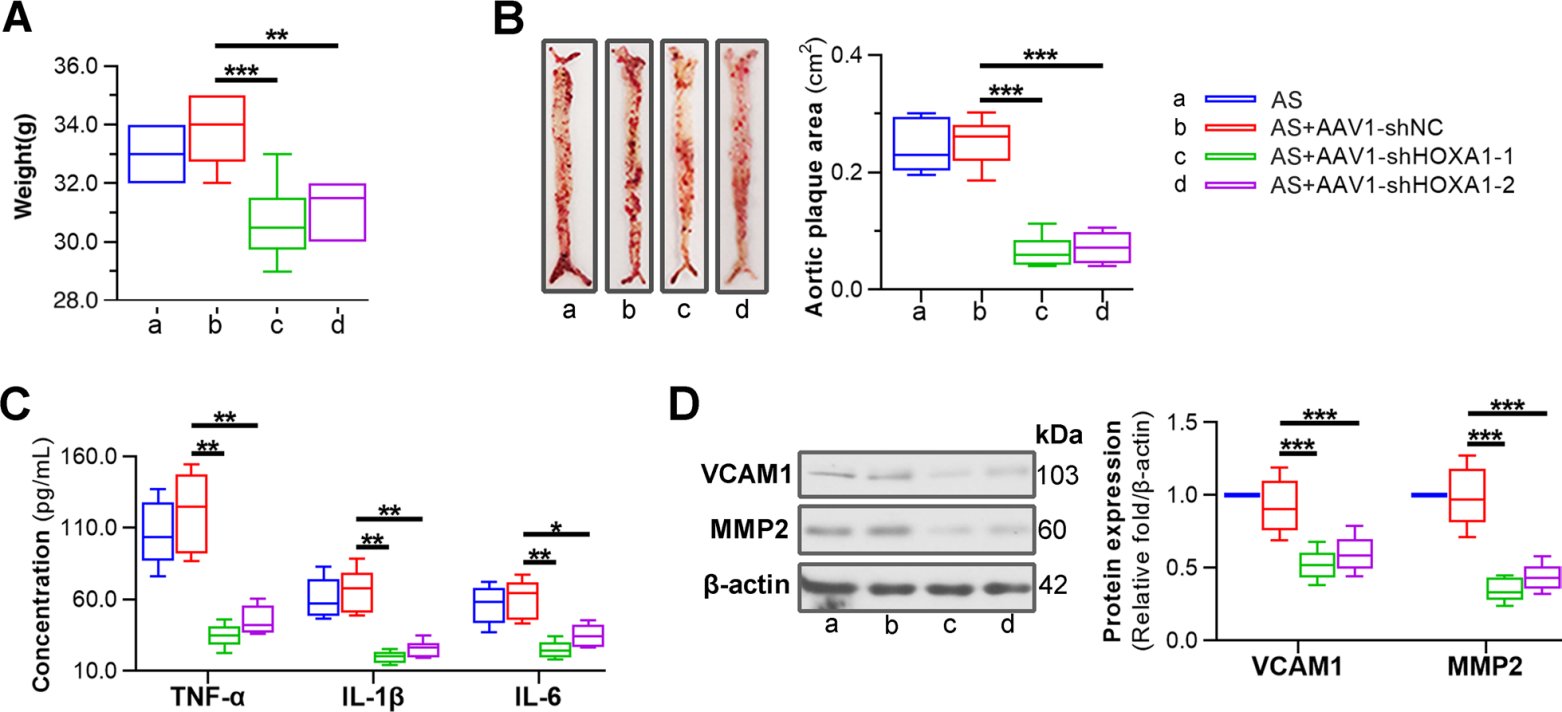
Silencing of HOXA1 reduces weight gain, aortic lesions, and inflammation in HFD-fed ApoE^−/−^ mice. **A** In vivo loss-of-function study was performed in AS model (HFD-fed ApoE^−/−^) mice by transduction of AAV1-shNC, AAV1-shHOXA1-1 or AAV1-shHOXA1-2 via tail vein. The final wight in 12 weeks after mice receiving HFD feeding was recorded. **B** Oil red-O staining was performed on entire aortas and the positive regions (plaques) were quantified. **C** ELISA for TNF-α, IL-1β and IL-6 levels in thoracic aortas of mice in each group. **D** Western blot assay for VCAM1 and MMP2 expression in thoracic aorta. Data are expressed as mean ± SD (6 animals/group). Ordinary one-way ANOVA followed by Tukey's multiple comparison test was used to calculate the *P* value in panel **A**, **B** and **D**. Brown-Forsythe and Welch ANOVA followed by Games-Howell's multiple comparisons test was used to calculate the *P* value in panel **C**. ^*^P < 0.05, ^**^P < 0.01, ^***^P < 0.001

In AS mice with transduction of AAV-1 shHOXA1-1/2 vectors, the final weights were significantly decreased compared to that with AAV-1 shNC vector (Fig. 2A). Histopathological examinations were then performed to show the change of plaques on the entire arterial trunk. The results presented that HOXA1 depletion by AAV1-shHOXA1-1/2 significantly decreased the aortic plaque area in AS mice (Fig. 2B). Moreover, there is accumulating evidence that AS is a chronic inflammatory disease (Wolf and Ley 2019). AS-related inflammation is mediated by pro-inflammatory cytokines, signalling pathways, lipids, enzymes, and adhesion molecules. We then detected the expression of several key inflammatory molecules including VCAM1, MMP2, TNF-α, IL-1β and IL-6. We observed that HOXA1 depletion significantly decreased the levels of these factors in AS mice (Fig. 2C, D).

### Silencing of HOXA1 in the liver attenuates AS-associated lipid metabolic disorder

**Fig. 3:**
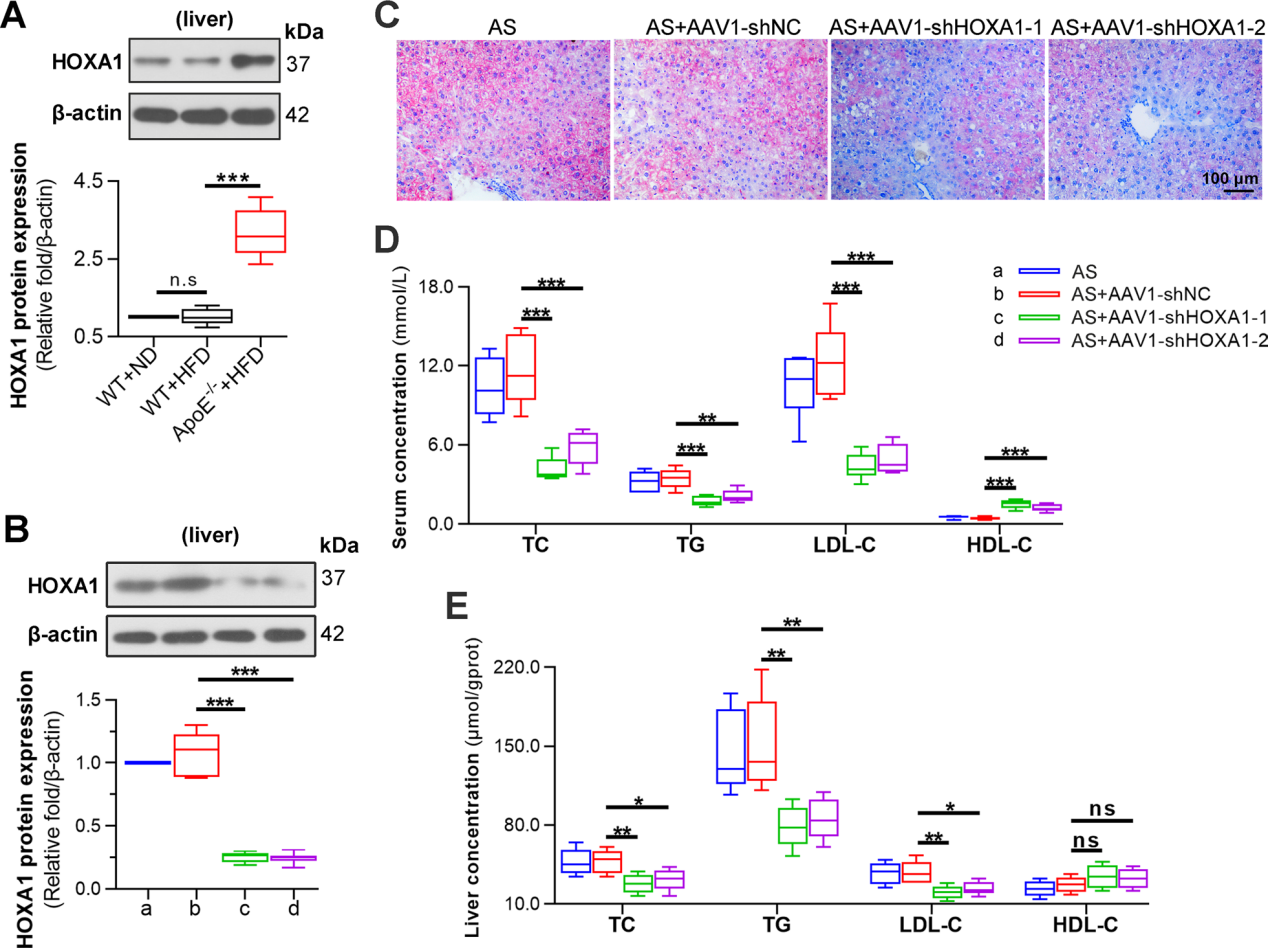
Silencing of HOXA1 in the liver attenuates AS-associated lipid metabolic disorder. **A** Western blot for HOXA1 expression in the liver of wild-type mice with ND, wild-type mice with HFD, or ApoE^−/−^ mice with HFD. **B** Western blot for HOXA1 expression in the liver of HFD-fed ApoE^−/−^ mice without or with in vivo transduction of AAV1-shNC, AAV1-shHOXA1-1 or AAV1-shHOXA1-2. **C** Oil red-O staining for the liver of mice in each group. Scale bar = 100 μm (200 ×). **D** Levels of serum lipids TC, TG, LDL-C, and HDL-C in each group. **E** Levels of TC, TG, LDL-C, and HDL-C in the liver of each group. Data are expressed as mean ± SD (6 animals/group). Two tailed unpaired t-test was used to calculate the *P* value in panel **A**. Ordinary one-way ANOVA followed by Tukey's multiple comparison test was used to calculate the *P* value in panel **B**, **D**, **E**. ^*^P < 0.05, ^**^P < 0.01, ^***^P < 0.001, ns: no significance.

Lipid metabolic disorder is one of the key features and incentives of AS pathogenesis (Poznyak et al. 2020). We next detected several parameters of lipid homeostasis. It turned out that the serum levels of TC, TG and LDL-C were notably decreased, whereas HDL-C level was increased after HOXA1 inhibition (Fig. 3D). Since changes of serum lipid parameters are mainly mediated by the liver (Zhang et al. 2022), and we used a systemic viral intervention system via tail vein in this work, we suspected that HOXA1 may have a direct effect on lipid synthesis in the liver. So we examined the HOXA1 expression in liver. It was shown that HOXA1 expression was significantly higher in the liver of AS mice (Fig. 3A). AAV1-mediated HOXA1-shRNA delivery also decreased HOXA1 expression in the liver (Fig. 3B). Oil-red O staining presented that the AS-associated lipid accumulation in the liver was inhibited by AAV1-shHOXA1-1/2 transduction (Fig. 3C). Furthermore, the hepatic levels of TC, TG and LDL-C were notably decreased by HOXA1 knockdown, whereas HDL-C level was increased slightly but with no significance (Fig. 3E). These findings implicated that the effect of HOXA1 on AS may involve the changes on hepatic lipid metabolism.

### Silencing of HOXA1 reduces atherosclerotic lesions by inhibiting cell apoptosis and macrophage-like phenotype switching of AS mice

**Fig. 4:**
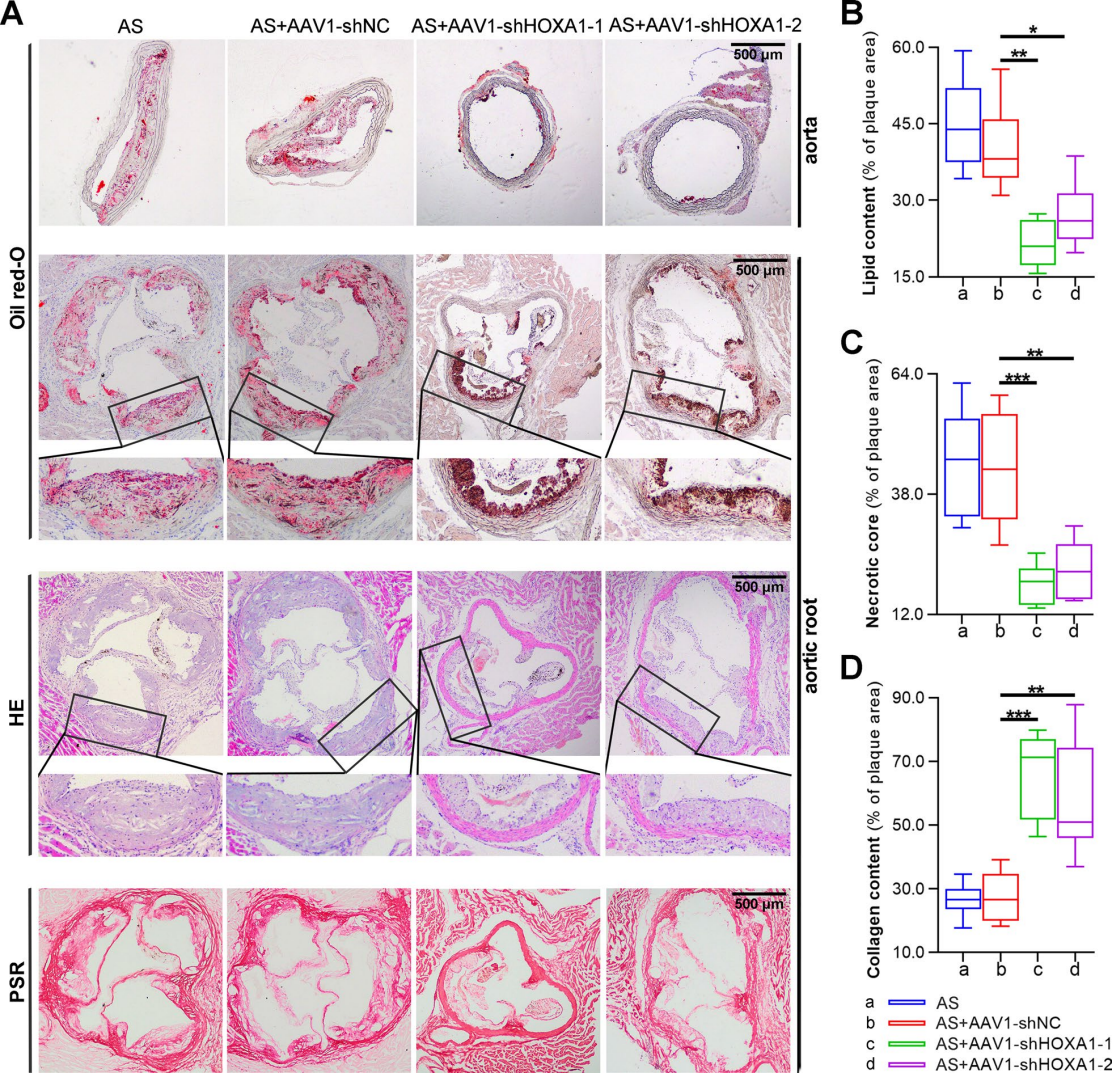
Silencing of HOXA1 reduces atherosclerotic lesions of AS mice. **A** Oil red-O, HE, and PSR staining for thoracic aorta or aortic root sections of mice in each group. Scale bar = 500 μm (40 ×); Quantification for lipid content (Oil red-O, panel **B**), necrotic core area (HE, panel **C**) and collagen content (PSR, panel **D**). Data are expressed as mean ± SD (6 animals/group). Ordinary one-way ANOVA followed by Tukey's multiple comparison test was used to calculate the *P* value in panel **B**–**D**. ^*^P < 0.05, ^**^P < 0.01, ^***^P < 0.001

**Fig. 5:**
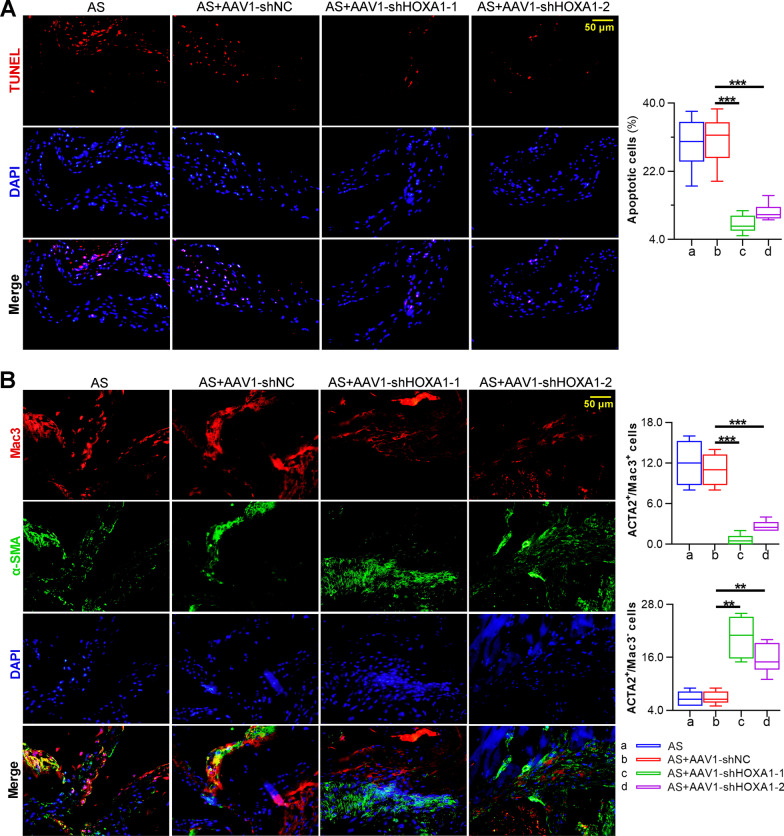
Silencing of HOXA1 inhibits apoptosis and reduces macrophage-like cells in plaques of AS mice. **A** TUNEL staining for plaque apoptosis and quantification for the positive staining regions. Sections were stained for TUNEL (red). DAPI (blue) was used for nucleus staining. Scale bar = 50 μm (400 ×). **B** Immunofluorescence staining for α-SMA and Mac3 in plaques and quantification for the α-SMA^+^/Mac3^+^ and α-SMA^+^/Mac3^−^ regions. Sections were co-stained for macrophage phenotype marker Mac3 (red) and smooth muscle cell marker α-SMA (green). DAPI (blue) was used for nucleus staining. Scale bar = 50 μm (400 ×). Data are expressed as mean ± SD (6 animals/group). Ordinary one-way ANOVA followed by Tukey's multiple comparison test was used to calculate the *P* value in panel **A**. Brown-Forsythe and Welch ANOVA followed by Games-Howell's multiple comparisons test was used to calculate the *P* value in panel **B**. ^**^P < 0.01, ^***^P < 0.001

To further investigate the effect of HOXA1 silencing on the formation of atherosclerotic lesions***,*** histopathological examinations were performed. The paraffin-embedded sections of thoracic aorta were subjected to oil red-O staining, and those sections of aortic root were subjected to oil red-O staining, HE staining, and PSR staining to determine lipid content, necrotic cores and collagen content in the plaque respectively (Fig. 4A). Quantitative analysis revealed that AAV1-mediated HOXA1 depletion in AS mice markedly decreased lipid content and necrotic core, whereas increased collagen content in the aortic root (Fig. 4B–D). Moreover, it is well acknowledged that the reduced apoptotic cell clearance links with plaque development (Thorp 2010). Herein, TUNEL staining was performed to detect apoptotic cells in the aortic root. Quantitative analysis showed that the apoptotic cells in the plaques of aortic root were significantly reduced after HOXA1 depletion (Fig. 5A). Also, phenotype switching of SMC is pivotal in atherosclerotic lesions (Pan et al. 2020). By double-immunostaining with SMC marker α-SMA and macrophage marker Mac3, we observed a significant reduction in macrophage-like cell number, and a significant increase in VSMC number within plaques of aortic roots in AS mice with AAV1-shHOXA1 injection (Fig. 5B), indicating that HOXA1 may be involved in the VSMC-to-macrophage transdifferentiation in AS.

### Silencing of HOXA1 suppresses RelA and KLF4 expression of AS mice

**Fig. 6:**
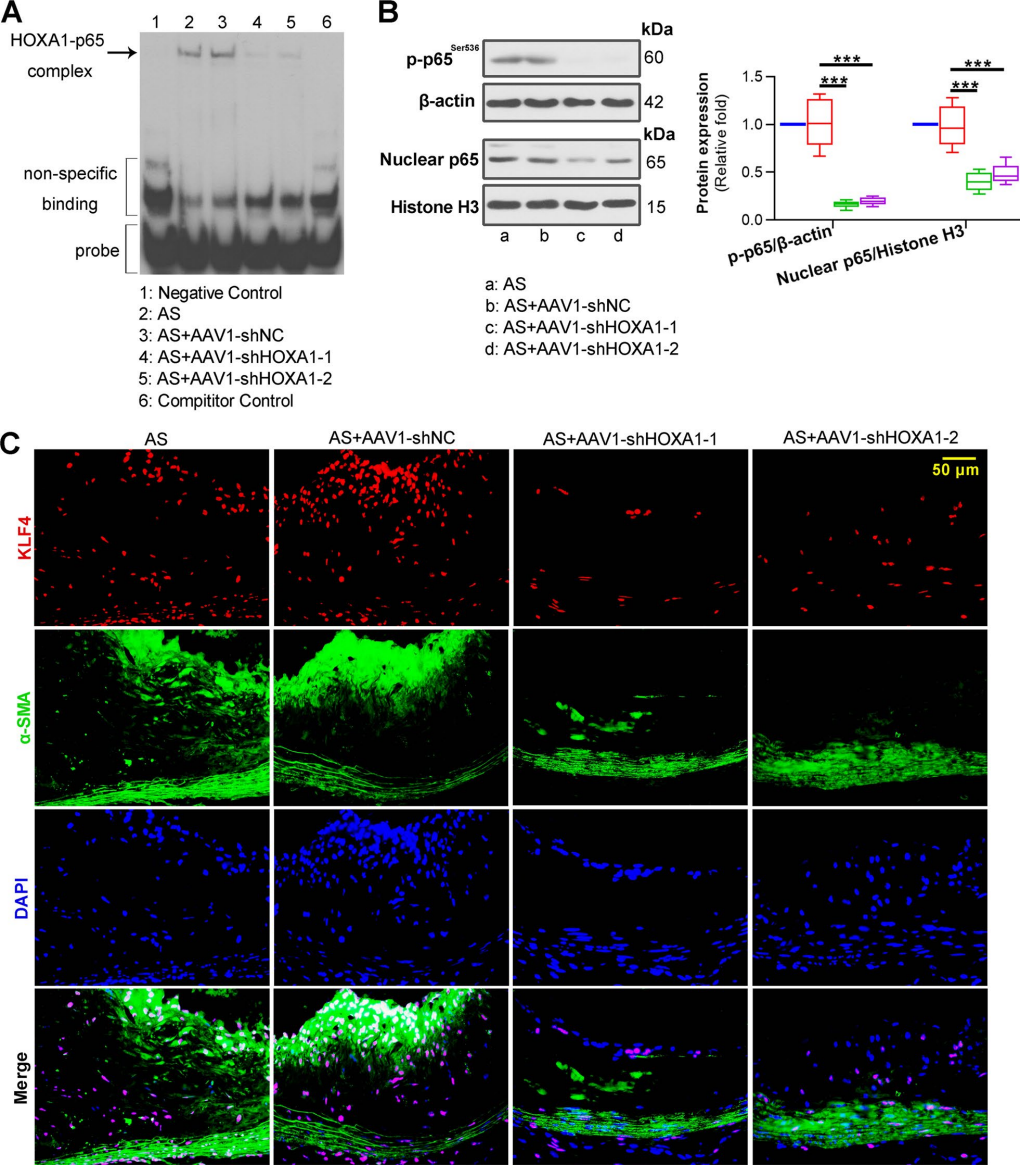
Silencing of HOXA1 inhibits NF-κB p65 and *KLF4* expression of AS mice. **A** EMSA for HOXA1 binding to NF-κB *RelA* promoter in plaques of mice in each group. **B** Western blot assay for phospho-p65^ser536^ and nuclear p65 expression in plaques. **C** Immunofluorescence double staining for KLF4 (red) and a-SMA (green) in plaques of the aortic root. DAPI (blue) was used for nucleus staining. Scale bar = 50 μm (400 ×). Data are expressed as mean ± SD (6 animals/group). Ordinary one-way ANOVA followed by Tukey's multiple comparison test was used to calculate the *P* value in panel **B**. ^***^P < 0.001

Due to the importance of nuclear factors NF-κB *RelA* (p65) and *KLF4* in AS progression, we investigated the potential of their involvement in HOXA1 regulatory mechanism. EMSA results exhibited that the DNA-binding activities of NF-κB were significantly inhibited in AAV1-shHOXA1-1/2 groups (Fig. 6A). Western blot assay showed that HOXA1 depletion inhibited the phosphorylation and nuclear expression of NF-κB p65 (Fig. 6B). Further, immunofluorescence double staining displayed that inhibition of HOXA1 suppressed the KLF4 expression in the SMCs of aortic root plaques in AS mice (Fig. 6C).

### Silencing of HOXA1 suppresses lipid accumulation, VSMC-to-macrophage transdifferentiation, and inflammation in POV-PC treated VSMCs

**Fig. 7:**
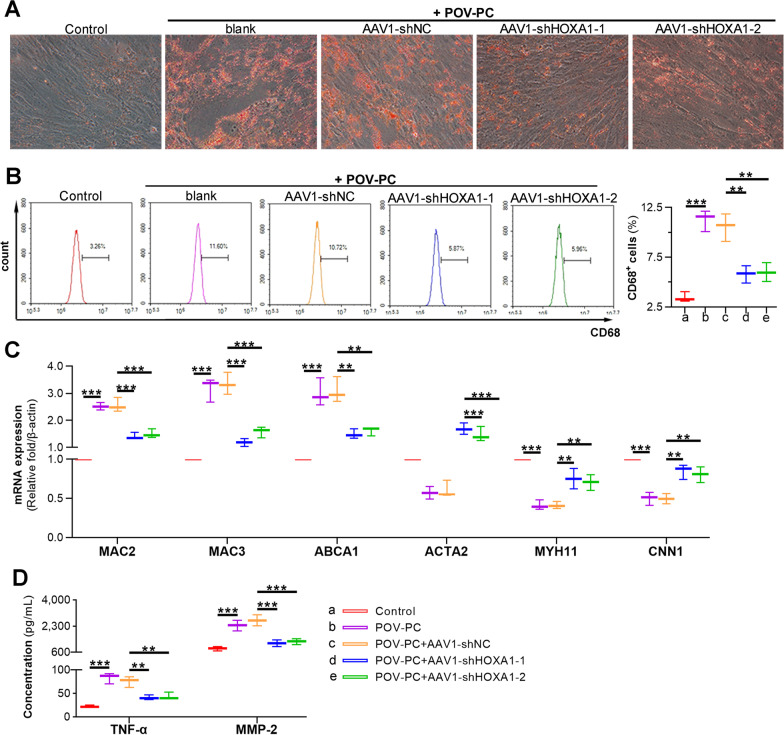
Silencing of HOXA1 suppresses lipid accumulation, VSMC-to-macrophage transdifferentiation, and inflammation of POV-PC treated VSMCs. POV-PC treated VSMCs were applied as in vitro model of AS. Loss-of-function investigation was performed by transduction of AAV1-shNC, AAV1-shHOXA1-1 or AAV1-shHOXA1-2. **A** Oil-red O staining for lipids in POV-PC treated VSMCs. **B** Flow cytometry analysis and quantification for CD68^+^ cells. **C** Real-time qPCR assay for *MAC2, MAC3, ABCA1*, *ACTA2, MYH11* and *CNN1* expression. **D** ELISA for TNF-α and MMP-2 levels. Data are expressed as mean ± SD (n = 3). Ordinary one-way ANOVA followed by Tukey's multiple comparison test was used to calculate the *P* value in panel **B**–**D**. ^**^P < 0.01, ^***^P < 0.001

Oxidized LDL (ox-LDL) affects AS progression through acting on multiple cells including VSMCs (Brito et al. 2009). We stimulated VSMCs with POV-PC in vitro, followed with AAV1 mediated gene transferring. Successful silencing of HOXA1 was observed at both mRNA and protein levels (Additional file 1: Fig. S1C, D). Results of oil-red O staining presented an increased accumulation of cellular lipid in VSMCs post POV-PC stimulation, whereas HOXA1 depletion effectively reduced the raised lipid content (Fig. 7A). Next, we investigated the effect of HOXA1 depletion on the VSMC-to-macrophage transdifferentiation. Macrophage marker CD68 was used to label macrophage-like VSMCs and flow cytometry was performed to analyse the ratio of CD68^−^positive cells. It turned out that the ratio of CD68^+^ cells were markedly increased in POV-PC treated VSMCs compared to control cells. Reversely, less CD68^+^ VSMCs were observed in AAV1-shHOXV1 intervention groups compared to AAV1-shNC intervention group (Fig. 7B). Additionally, we detected the expression of macrophage markers *MAC2, MAC3, ABCA1* and SMC markers *ACTA2* (α-SMA)*, MYH11, CNN1* using real-time qPCR. The results showed that POV-PC stimulation significantly increased *MAC2, MAC3, ABCA1*, but decreased *ACTA2* mRNA levels in VSMCs. Reversely, HOXA1 depletion reduced *Mac3* and increased *ACTA2, MYH11, CNN1* mRNA levels, suggesting that HOXA1 depletion suppressed the VSMC-to-macrophage transdifferentiation in POV-PC treated VSMCs (Fig. 7C). Furthermore, the levels of two representative inflammatory molecules TNF-α and MMP-2 were also found increased in VSMCs after POV-PC stimulation, which was partially reverted by inhibition of HOXA1 (Fig. 7D).

### HOXA1 directly binds to RelA promoter and KLF4 promoter to regulate their expression

**Fig. 8:**
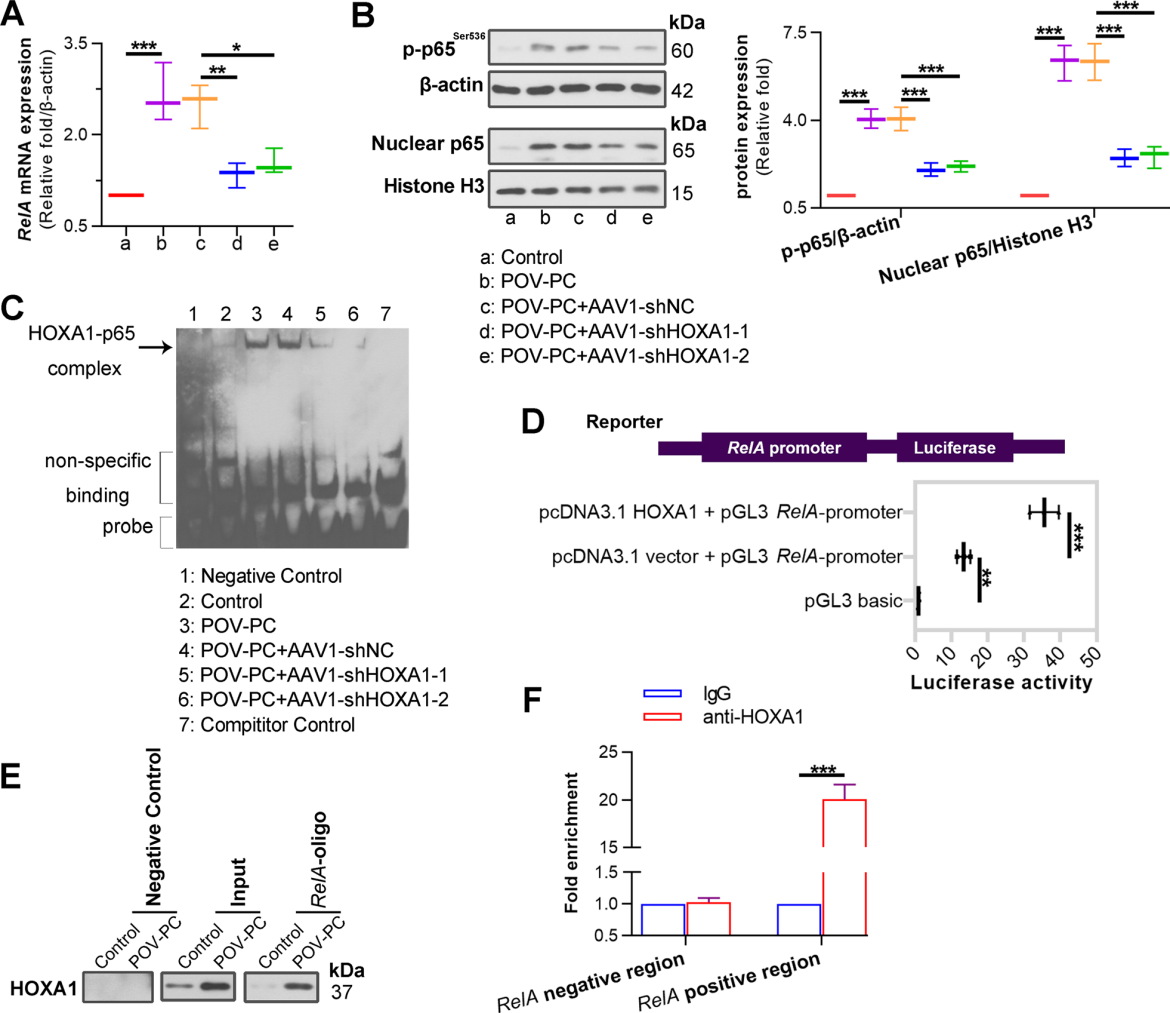
HOXA1 transcriptionally activates *RelA*. **A** Real-time qPCR assay for NF-κB *RelA* expression in POV-PC treated VSMCs. **B** Western blot assay for phospho-p65^ser536^ and nuclear p65 expression. **C** EMSA for HOXA1 binding to NF-κB *RelA* promoter in POV-PC treated VSMCs. **D** 293 T cells were co-transfected with a GFP-HOXA1 expression vector and a *RelA*-promoter reporter plasmid for 72 h. The cell lysates were prepared for luciferase assay. **E** The cell lysates of VSMCs were subjected to oligonucleotide pull-down assay with biotinylated double-stranded oligonucleotides containing *RelA*-promoter as probe. Nonspecific oligonucleotide sequences were applied as a negative control. The retrieved protein was collected and analysed by Western blot with anti-HOXA1 antibody. **F** ChIP-qPCR assay was performed in VSMCs with anti-HOXA1 antibody and non-immune IgG was used as an internal control. Immunoprecipitated DNA was amplified by PCR using *RelA*-promoter primers. Negative regions (regions downstream of the *RelA*-promoter) was adopted as an amplification control. Data are expressed as mean ± SD (n = 3). Ordinary one-way ANOVA followed by Tukey's multiple comparison test was used to calculate the *P* value in panel **A**, **B**, **D**. Two tailed unpaired t-test was used to calculate the *P* value in panel **F**. ^*^P < 0.05, ^**^P < 0.01, ^***^P < 0.001

**Fig. 9:**
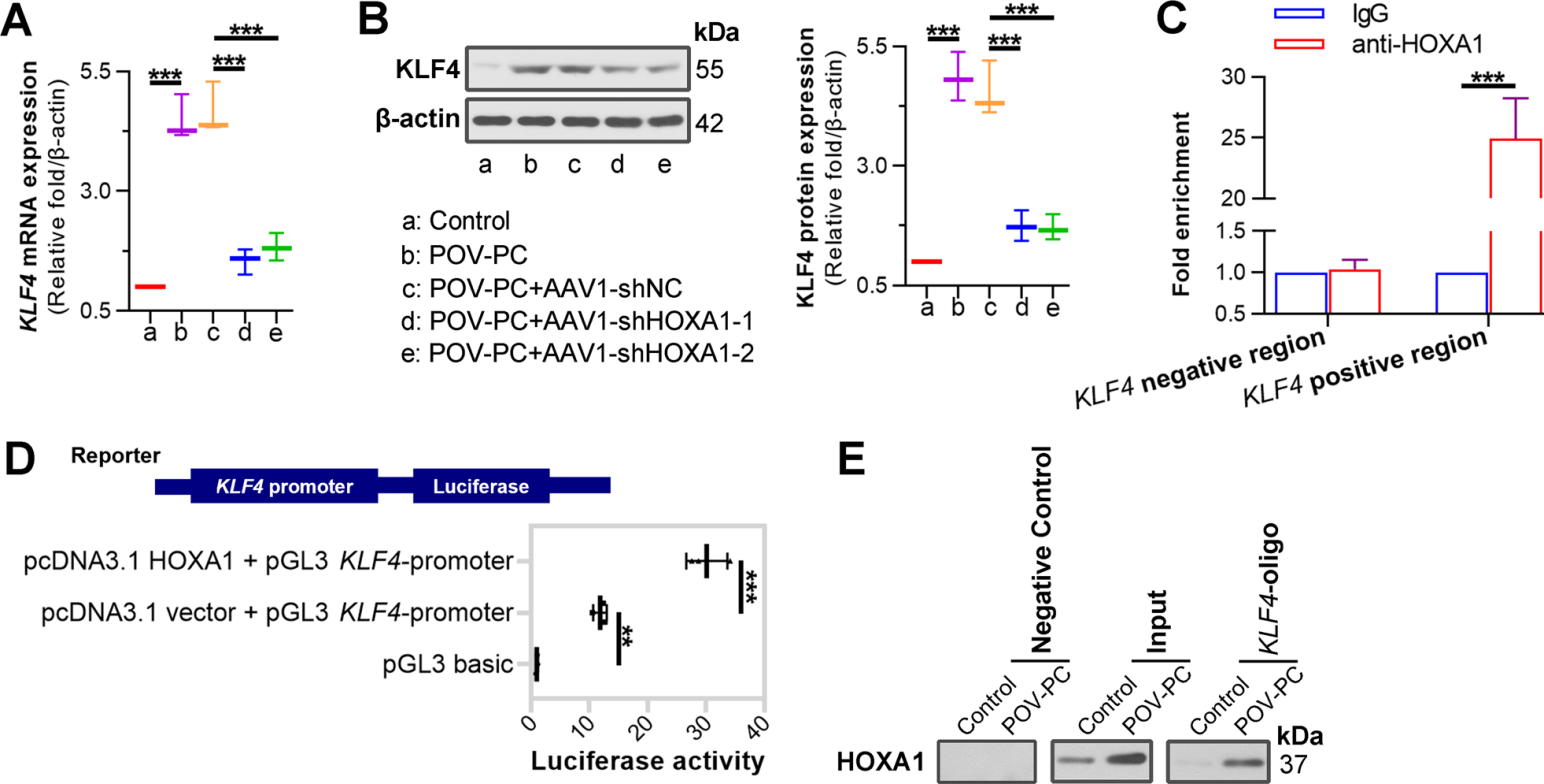
HOXA1 transcriptionally activates *KLF4*. **A** Real-time qPCR assay for *KLF4* expression in POV-PC treated VSMCs. **B** Western blot assay for KLF4 expression. **C** ChIP-qPCR assay was performed in VSMCs with anti-HOXA1 antibody and non-immune IgG was used as an internal control. Immunoprecipitated DNA was amplified by PCR using *KLF4*-promoter primers. Negative regions (regions downstream of the *KLF4*-promoter) was adopted as an amplification control. **D** 293 T cells were co-transfected with a GFP-HOXA1 expression vector and a *KLF4*-promoter reporter plasmid for 72 h. The cell lysates were prepared for luciferase assay. **E** The cell lysates of VSMCs were subjected to oligonucleotide pull-down assay with biotinylated double-stranded oligonucleotides containing *KLF4*-promoter as probe. Nonspecific oligonucleotide sequences were applied as a negative control. The retrieved protein was collected and analysed by Western blot with anti-HOXA1 antibody. Data are expressed as mean ± SD (n = 3). Ordinary one-way ANOVA followed by Tukey's multiple comparison test was used to calculate the *P* value in panel **A**, **B**, **D.** Two tailed unpaired t-test was used to calculate the *P* value in panel **C**. ^**^P < 0.01, ^***^P < 0.001

Mechanically, we observed that POV-PC treatment increased the levels of *RelA* mRNA (Fig. 8A), phosphorylated-p65 protein (Fig. 8B), nuclear p65 protein (Fig. 8B), *KLF4* mRNA (Fig. 9A) and KLF4 protein (Fig. 9B) in VSMCs, whereas inhibition of HOXA1 decreased these levels. According to the prediction by JASPAR (http://jaspar.genereg.net/), it was inferred that HOXA1 could directly bind to the *RelA* promoter, and also the *KLF4* promoter. To verify this inference, we used biological testing methods to evaluate the transcriptional regulation of HOXA1 on *RelA* and *KLF4* expression. EMSA results showed that HOXA1 depletion notably reduced the elevated protein-DNA complex content post POV-PC treatment (Fig. 8C). Luciferase reporter assays revealed that luciferase activity was significantly increased in 293 T cells co-transfected with HOXA1 overexpression plasmid and *RelA* promoter vector compared to cells transfected with empty plasmid and *RelA* promoter vector (Fig. 8D). Same phenomenon was observed with co-transfection of *KLF4* promoter vector (Fig. 9D). Besides, oligonucleotide pull-down and ChIP-qPCR assays suggested that POV-PC treatment notably increased the HOXA1-*RelA* promoter binding ability (Fig. 8E, F), as well as the HOXA1-*KLF4* promoter binding ability (Fig. 9C, E). These data implicated that HOXA1 transcriptionally regulated *RelA* and *KLF4* expression.

### HOXA1 is involved in the pathological manifestations of VSMCs via RelA and KLF4

**Fig. 10:**
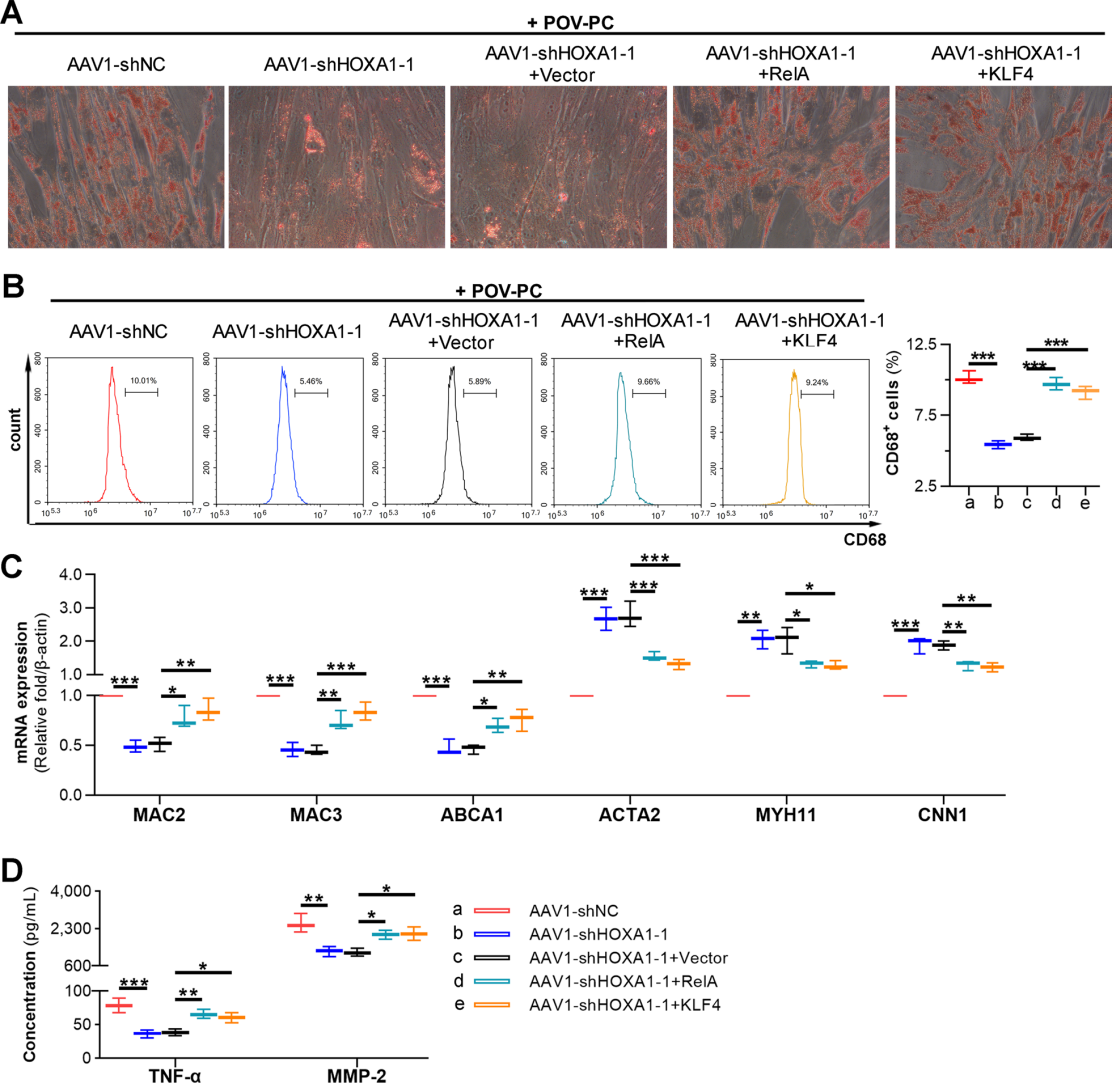
HOXA1 affects lipid accumulation, VSMC-to-macrophage transdifferentiation, and inflammation of POV-PC treated VSMCs via regulation of RelA and KLF4. The POV-PC treated VSMCs were infected with AAV1-shNC or AAV1-shHOXA1-1 together with RelA and KLF4 overexpression plasmid. **A** Oil-red O staining for lipids in POV-PC treated VSMCs. **B** Flow cytometry analysis and quantification for CD68^+^ cells. **C** Real-time qPCR assay for *MAC2, MAC3, ABCA1*, *ACTA2, MYH11* and *CNN1* expression. **D** ELISA for TNF-α and MMP-2 levels. Data are expressed as mean ± SD (n = 3). Ordinary one-way ANOVA followed by Tukey's multiple comparison test was used to calculate the *P* value in panel **B**–**D**. ^*^P < 0.05, ^**^P < 0.01, ^***^P < 0.001

To further investigate whether RelA and KLF4 work as downstream effectors of HOXA1, we treated VSMCs with RelA or KLF4 overexpression plasmid to rescue the effect of HOXA1 silencing under POV-PC stimulation. The overexpression efficiencies of RelA and KLF4 after transfection were verified by real-time PCR and western blot (Additional file 2: Fig. S2). Pathological manifestations of VSMCs were next examined by oil-red O staining, flow cytometry, real-time PCR and ELISA as described above. Both overexpression of RelA and KLF4 abolished the inhibitory effects of HOXA1 silencing on lipid accumulation, VSMC-to-macrophage transdifferentiation, inflammatory molecules secretion in POV-PC treated VSMCs (Fig. 10), implicating that HOXA1 is involved in the pathological manifestations of VSMCs via regulation of RelA and KLF4.

## Discussion

HOXA1, a member of the *HOX* family, is a type of transcription factor (Draime et al. 2018). Its overexpression in atherosclerotic lesions has been previously reported by our lab (Han et al. 2019), but its role in AS has not been well-defined. In this work, we reveal for the first time that HOXA1 is a contributor to AS. Our findings experimentally delineate that the AAV1-mediated depletion of HOXA1 reduces atherosclerotic lesions, and one of the key mechanisms is that HOXA1 silencing suppresses the pathological manifestations of VSMCs. Possible molecular mechanisms may be related to the HOXA1-dependent regulation of *RelA* and *KLF4*.

AS originates as an innate immune response to the retained and modified cholesterol-rich lipoproteins in the intima of vessel wall, recruiting inflammatory cells from the circulation (Zimmer et al. 2016). As well known, rapid weight gain and lipid metabolism alterations in adulthood frequently lead to an increased risk of cardiometabolic disorders. Treatment of weight and lipid disorders remains the common strategy to attenuate AS (Li et al. 2017; Zhou et al. 2018). In our study, when HOXA1 expression was inhibited, the rapid weight gain and abnormal lipid metabolism were attenuated in HFD-induced AS mice. The reason why HOXA1 silencing could inhibit the body weight and serum lipids is worthy of investigation. Liver is the principal site for lipoprotein synthesis and metabolism in the circulation (Luoma 1988). HOXA1 has been reported to play an oncogenic role in hepatocellular carcinoma (Elfiky et al. 2021; Zha et al. 2012). Intriguingly, in the liver of HFD-fed ApoE^−/−^ mice, we observed HOXA1 was also raised. Due to the hepatic addictiveness of AAV1, it is possible that the AAV1-mediated HOXA1-shRNA delivery system also works at the liver. Results of western blot confirmed this conjecture. Besides, the imbalanced lipid metabolism in the liver was also mitigated by AAV1-mediated HOXA1 silencing in AS mice, implicating the effect of HOXA1 on AS may involve changes on the hepatic lipid metabolism.

Another characteristic feature of atherosclerotic lesions is the formation of plaques (Králová et al. 2014). In an atherosclerotic plaque, there are extracellular lipid particles, foam cells, dead cells and debris accumulating in the intima of vascular walls, forming a necrotic core. The necrotic core is surrounded by a layer of collagen-rich matrix and SMCs covered by endothelial cells (Vergallo and Crea 2020). Oxidized lipids, accumulation of foam cells and apoptotic cells, and enhancement of inflammation would accelerate the progression of an atherosclerotic plaque. The plaques may lead to many deleterious consequences including plaque rupture, vascular remodelling and necrosis (Kockx and Herman 1998; Sullivan 2007). In this work, the AS mice with HOXA1 depletion presented less plaques in the artery. Also, the plaques exhibited a reduction in lipid content and necrotic core area, as well as an increase in collagen content. Moreover, the inflammatory and apoptotic cells in the plaque were significantly inhibited by HOXA1 silencing.

Cells that participate in atherosclerotic plaque formation include VSMCs, endothelial cells, macrophages, dendritic cells and regulatory T cells (Munjal and Khandia 2020). VSMCs are predominant in all stages and play a vital role in AS progression. Loss of VSMCs causes fibrous cap thinning and leads to necrotic core formation and calcification. Ox-LDL and pro-inflammatory cytokines can induce VMSC injury and even apoptosis (Grootaert et al. 2018). With regard to these events, it is meaningful to attenuate the oxidized lipids-induced VSMC dysfunction in AS. In the POV-PC (a type of ox-LDL) treated VSMCs, we observed that silencing of HOXA1 reduced the POV-PC induced lipid accumulation and inflammatory response, suggesting a mechanism of HOXA1 in regulating AS is the improvement of VSMCs dysfunction.

Following the “damage response” and “vulnerable plaque” hypotheses, the contractile VSMCs undergo phenotypic switch into synthetic VSMCs, producing extracellular matrix to stabilize the plaques. However, recent data from lineage-tracing and transcriptomic studies have pointed out the view that VSMCs reduce contractile markers during AS progression whilst adopting multiple alternative phenotypes, including phenotypes resembling foam cells and macrophages, which might be detrimental (Basatemur et al. 2019; Grootaert and Bennett 2021). Data from human (Allahverdian et al. 2014) and mouse plaques (Feil et al. 2014) confirm that a subset of VSMCs that migrate from the medial layer into the plaque intima lose specific markers of contractile phenotype, such as smooth muscle protein 22 alpha (SM22α) and ACTA2 (α-SMA) (Oh et al. 2020), and obtain macrophage specific markers such as CD68 or Mac-2 (Giannotti et al. 2019). These VSMC-derived macrophage-like cells are sensitive to lipid content and cholesterol loading, which allows them to engulf lipids, dead cells, and other substances perceived as danger signals and to secrete a plenty of harmful molecules (Tabas and Bornfeldt 2016). Notably, Feil et al. demonstrated that targeting VSMC-to-macrophage phenotypic switching might become a novel strategy for treating AS (Feil et al. 2014). In this work, we observed that HOXA1 silencing reduced the macrophage-like cells in atherosclerotic plaque, as well as inhibited the VSMC-to-macrophage transdifferentiation.

As a highly conserved transcription factor, HOXA1 plays diverse functional roles in differentiation and development by transcriptionally regulating multiple genes. By using bioinformatic analysis, we preliminarily inferred that HOXA1 may transcriptionally activate *RelA*. NF-κB is regarded as a proatherogenic factor in responding to harmful cellular stimuli, as it regulates a lot of proinflammatory molecules that link AS (Pateras et al. 2014). Taminiau et al. suggested a strong positive correlation between HOXA1 expression and TNF/NF-κB pathway (Taminiau et al. 2016). The activation of NF-κB by HOXA1 occurs upstream of NF-κB nuclear translocation. Post-translational modifications, especially phosphorylation on the RelA/p65 sub-unit, are critical for the cytoplasmic to nuclear translocation of NF-κB. In the current study, we experimentally demonstrated that NF-κB *RelA* is a direct transcription target of HOXA1. HOXA1 influences the phosphorylation and nuclear translocation of NF-κB. The exact mechanism of the latter events has not been elucidated, but one hypothesis is that HOXA1 induces a positive feedback of pro-inflammatory cytokines to activate the NF-κB signalling. We also found that HOXA1 has binding sites on the promoter of *KLF4* by bioinformatic analysis. The KLF4-dependent phenotypic modulation of VSMCs to macrophage-like cells is proved to be critical in atherosclerotic plaque pathogenesis (Rosenfeld 2015; Shankman et al. 2015). With experimental evidence, we illustrated KLF4 is a direct transcription target of HOXA1 as well. Rescue experiments confirmed that HOXA1 regulates the pathological manifestations of VSMCs via NF-κB RelA and KLF4. Intriguingly, Ding et al. discovered that pharmacological or genetic inhibition of NF-κB decreases the expression of KLF4 in VSMCs from AMPKα2^−/−^ mice (Ding et al. 2016). The positive feedback relationship in NF-κB and KLF4 gives a possible explanation for the molecular mechanism of HOXA1 in regulating AS progression.

Regrettably, this study has limitations. First, we only examined HOXA1 in VSMCs. The expression pattern and function of HOXA1 in other AS-associated cells (such as endothelial cells and resident macrophages) remain uncertain. Second, the HOXA1 intervention in mice was global. To better clarify HOXA1’s role in VSMCs, it would get great significance if the intervention could be done in VSMCs. Third, the pathological observations in this study focus on the aortic root. However, blood flow is also a critical factor in AS pathogenesis, and the influence of shear force generated by blood flow is mainly reflected in the aortic arch. The current topic lacks the research and discussion on the aortic arch. Fourth, the HOXA1’s effects on KLF4 and p65 should be discussed specifically in VSMCs, but not in an overall artery without distinguishing cell types. Nevertheless, our results are still attractive and promising as we propose a new molecular mechanism of AS progression: HOXA1 could influence VSMCs alterations by regulating NF-κB and KLF4. HOXA1’s function in other cell lineages (such as endothelial cells or macrophages), or other parts of aorta (such as aortic arch), as well as other regulatory mechanisms, are worthy of future research.

## Conclusion

**Fig. 11:**
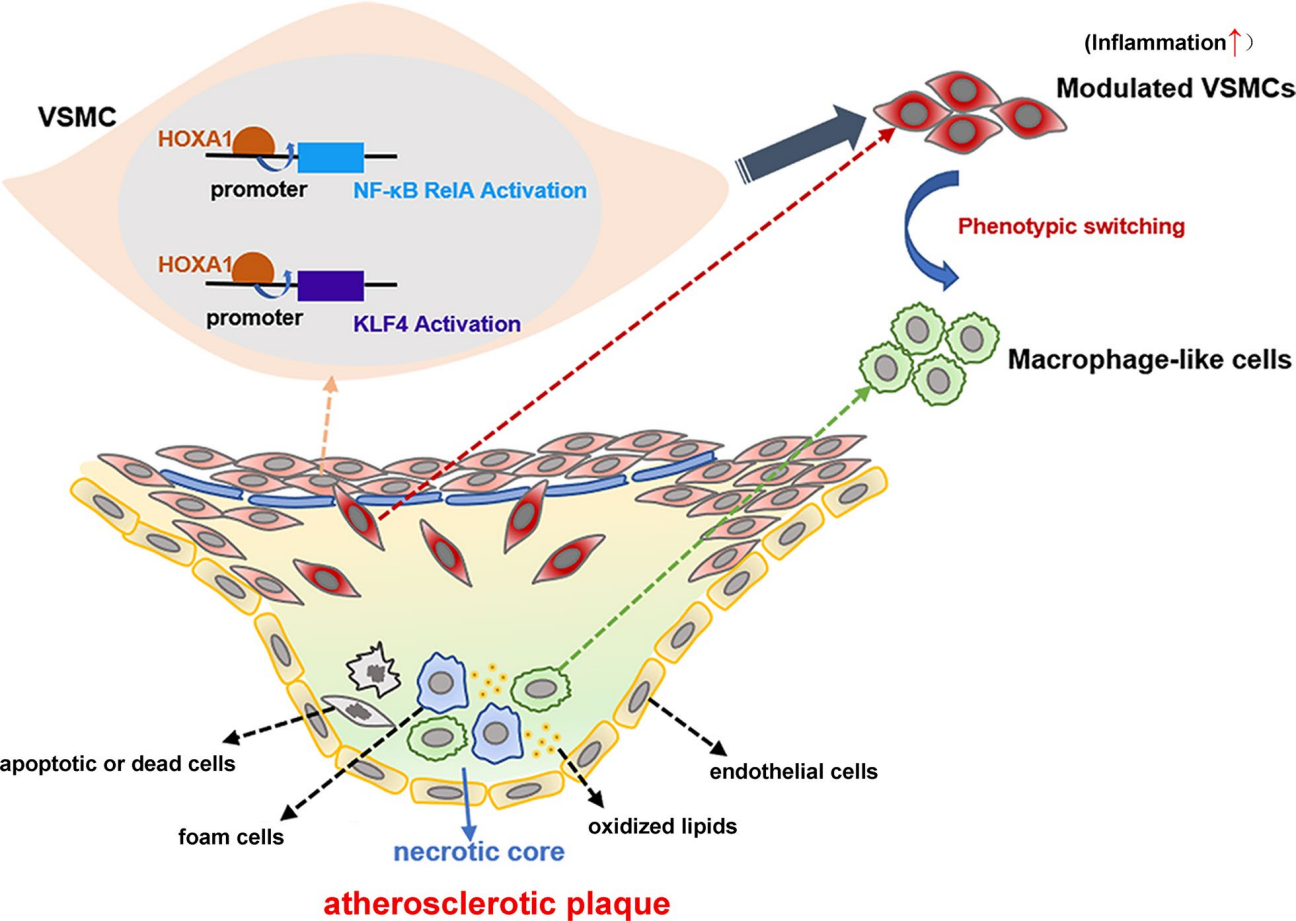
The mechanism diagram revealing the regulation of HOXA1 on atherosclerotic lesions. Under pathological conditions, HOXA1 transcriptionally activates the key transcription factors NF-κB *RelA* and *KLF4* in VSMCs, which drives the transformation of VSMCs into modulated VSMCs. Then the modulated VSMCs undergo phenotypic conversion and transdifferentiate into macrophage-like cells. Accumulation of these macrophage-like cells and other risk factors such as lipids, foam cells, and necrotic cells, contributes to the formation of atherosclerotic plaques

In conclusion, we show for the first time that HOXA1 participates in atherosclerotic lesions by regulating the VSMCs plasticity. Especially, we confirm that HOXA1 transcriptionally activates NF-κB *RelA* and *KLF4* to participate in the pathological manifestations of VSMCs. The mechanism diagram revealing the regulation of HOXA1 on atherosclerotic lesions formation is shown in Fig. 11. Our data suggest that HOXA1 appears to become a promising target for treating AS.

## Supplementary Information

**Additional file 1: Fig. S1.** Depletion of HOXA1 in AS mice and VSMCs with AAV1-shRNA technology. A Real-time qPCR and B western blot assays for HOXA1 expression in thoracic aortas of HFD-fed ApoE^−/−^ mice without or with in vivo transduction of AAV1-shNC, AAV1-shHOXA1-1 or AAV1-shHOXA1-2. Data are expressed as mean ± SD. C Real-time qPCR and D western blot assays for HOXA1 expression in VSMCs without or with in vitro transduction of AAV1-shNC, AAV1-shHOXA1-1 or AAV1-shHOXA1-2. Data are expressed as mean ± SD. Ordinary one-way ANOVA followed by Tukey's multiple comparison test was used to calculate the P value in panel A-D. ^**^P < 0.01, ^***^P < 0.001.

**Additional file 2: Fig. S2.** Overexpression of RelA and KLF4 in VSMCs. VSMCs were transfected with RelA overexpression plasmid, KLF4 overexpression plasmid, or blank vector for 24 h. A Real-time qPCR and B western blot assays for RelA expression in VSMCs after transfection. C Real-time qPCR and D western blot assays for KLF4 expression in VSMCs after transfection. Data are expressed as mean ± SD. Two tailed unpaired t-test was used to calculate the *P* value in panel A-D. ^***^P < 0.001.

## Data Availability

The datasets used and/or analysed during this study were accessed via the corresponding authors on reasonable request.

## Abbreviations

AAV-1: Adeno-associated-virus-1
ApoE−/−: Apolipoprotein E knockout
AS: Atherosclerosis
ChIP: Chromatin immunoprecipitation
ELISA: Enzyme-linked immunosorbent assay
EMSA: Electrophoretic mobility shift assay
HDL-C: High-density lipoprotein cholesterol
HFD: High fat diet
HOXA1: Homeobox A1
IL-1β: Interleukin-1β
IL-6: Interleukin-6
KLF4: Krüppel-like factor 4
LDL-C: Low-density lipoprotein cholesterol
MMP-2: Matrix metalloproteinase 2
Nuclear factor-κB: NF-κB
POV-PC: 1-Palmitoyl-2-(5-oxovaleroyl)-sn-glycero-3-phosphocholine
Real-time qPCR: Real-time quantitative polymerase chain reaction
shRNA: Short-hairpin RNA
TC: Total cholesterol
TG: Triglyceride
TNF-α: Tumour necrosis factor-α
VCAM1: Vascular cell adhesion molecule1
VSMC: Vascular smooth muscle cell
