# Supplementary figures and images for: HOXA1 participates in VSMC-to-macrophage-like cell transformation via regulation of NF-κB p65 and KLF4: a potential mechanism of atherosclerosis pathogenesis
Source: Mol Med. 2023 Aug 1;29:104. doi: 10.1186/s10020-023-00685-8 (PMC10394793; doi:10.1186/s10020-023-00685-8)

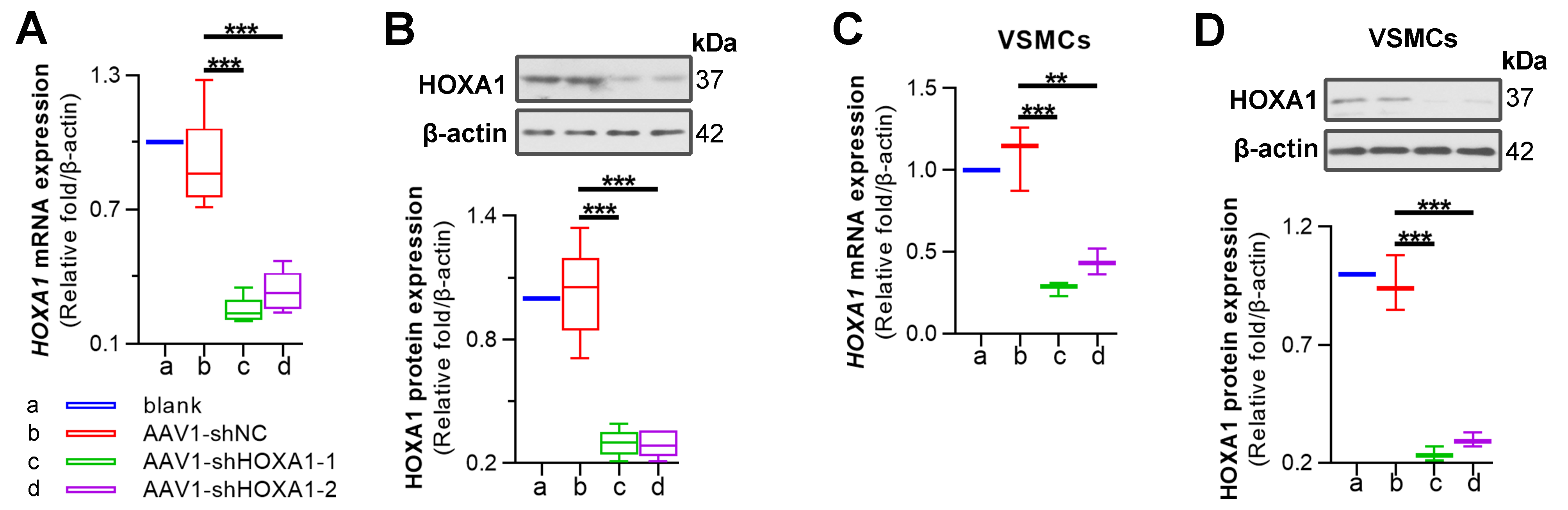

Supplement: Supplementary file 1 — Additional file 1: Fig. S1. Depletion of HOXA1 in AS mice and VSMCs with AAV1-shRNA technology. A Real-time qPCR and B western blot assays for HOXA1 expression in thoracic aortas of HFD-fed ApoE−/− mice without or with in vivo transduction of AAV1-shNC, AAV1-shHOXA1-1 or AAV1-shHOXA1-2. Data are expressed as mean ± SD. C Real-time qPCR and D western blot assays for HOXA1 expression in VSMCs without or with in vitro transduction of AAV1-shNC, AAV1-shHOXA1-1 or AAV1-shHOXA1-2. Data are expressed as mean ± SD. Ordinary one-way ANOVA followed by Tukey's multiple comparison test was used to calculate the P value in panel A-D. **P < 0.01, ***P < 0.001. [file 10020_2023_685_MOESM1_ESM.tif]

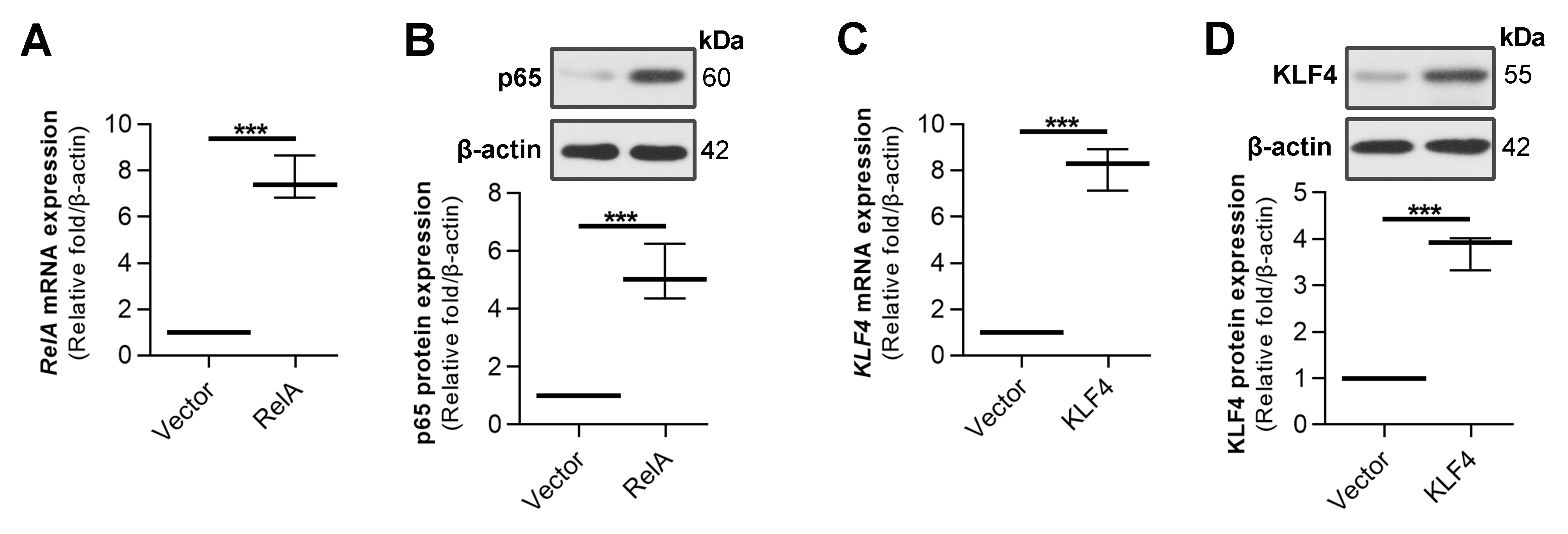

Supplement: Supplementary file 2 — Additional file 2: Fig. S2. Overexpression of RelA and KLF4 in VSMCs. VSMCs were transfected with RelA overexpression plasmid, KLF4 overexpression plasmid, or blank vector for 24 h. A Real-time qPCR and B western blot assays for RelA expression in VSMCs after transfection. C Real-time qPCR and D western blot assays for KLF4 expression in VSMCs after transfection. Data are expressed as mean ± SD. Two tailed unpaired t-test was used to calculate the P value in panel A-D. ***P < 0.001. [file 10020_2023_685_MOESM2_ESM.tif]
